# Dysfunction of B Cell Leading to Failure of Immunoglobulin Response Is Ameliorated by Dietary Silk Peptide in 14-Month-Old C57BL/6 Mice

**DOI:** 10.3389/fnut.2020.583186

**Published:** 2020-11-19

**Authors:** Sungwoo Chei, Hyun-Ji Oh, Kippeum Lee, Heegu Jin, Jeong-Yong Lee, Boo-Yong Lee

**Affiliations:** ^1^Department of Biomedical Sciences, CHA University, Pocheon, South Korea; ^2^Worldway Co., Ltd., Sejong, South Korea

**Keywords:** immunosenescence, senescence, aging, acid-hydrolyzed silk peptide, adaptive immunity, B cell

## Abstract

Anti-aging research suggests that immunosenescent cells can play deleterious roles in the immune system. Here, young (2 months old) and old (14 months old) C57BL/6 mice received a daily oral dose (100 or 750 mg/kg/day) of acid-hydrolyzed silk peptide (SP) for a period of 5 weeks. Mouse spleen, lymph node, and serum were analyzed to determine the immune homeostasis of SP by flow cytometry, Western blotting, ELISA, and qRT-PCR. The results suggest that SP ameliorates age-related dysfunction of T and B cells. Amelioration of B cell dysfunction improved the immunoglobulin response in aged mice. Taken together, the results suggest that SP restores immune homeostasis with respect to immunosenescent cells.

## Introduction

Age-related impairment of the immune system, known as immunosenescence, contributes to increased incidence of various diseases as the population ages (1). Immunosenescence is defined as a time-dependent functional decline/dysfunction of protective immunity, leading to a marked increase in chronic inflammatory disorders, infectious diseases, and autoimmune diseases (2–6). Excessive and aberrant accumulation of senescent cells can result in immune dysregulation or reduced immune competence in the host, resulting in onset and development of age-related diseases (7, 8). Immunosenescence is characterized by an unbalanced immune response and seemingly paradoxical alterations in all aspects of immunity in an aging-associated manner (9). It is essential to control immune homeostasis to balance the declining immune response in the aged. Thus, anti-aging research has focused on the deleterious role played by immunosenescent cells in the immune system (10, 11).

The immune system has an innate and an adaptive arm. T and B lymphocytes are central to the adaptive immune system (12, 13). T lymphocytes are most affected by aging, showing impaired effector function and reduced formation of T memory cells (13). There are two major types of T cell: the helper T cell and the cytotoxic T cell. The former, as the name suggests, help other immune cells to fight disease, whereas the latter actively kill infected cells and tumor cells (14). Antigen-specific helper T cells interact with antigen-specific B cells, leading to B cell expansion and differentiation.

B lymphocytes are also affected by aging, which leads to reduced effectiveness of the antibody response (15). B cells play central roles in establishing and maintaining protective immunity by producing antibodies and/or presenting antigens (16). There are three B lymphocyte subsets: B1, B2, and regulatory. B2 cells give rise to two mature subsets: mature follicular (FO) and marginal zone (MZ) B cells. Cells survive to join the major pre-immune B cells such as mature FO and MZ cells (17). FO B cells populate the follicles in the spleen and lymph nodes, whereas MZ B cells are important for host defense against circulating blood-borne pathogens (18). The immunoglobulins produced by B cells eliminate autoantigens, production of which increases with age, by removing apoptotic cells (19).

Cellular apoptosis plays an important role in aging. Senescent cells become resistant to apoptosis because they express high levels of anti-apoptotic genes such as BCL-2 or BCL-XL (20). In addition, expression of pro-apoptotic genes Bax, BAK, BID, and PUMA is associated with senescence (21). The BCL-2 family is essential for the survival of senescent cells (22). Genes such as p21 and p53, which induce cell cycle arrest, are markers in senescent cells (23).

To gain insight into the role of the silk peptide (SP) in immunosenescence, we examined its phenotypic and functional effects through adaptive immunity in young and aged mice. Consistent with immune dysfunction with aging, we found that aged mice had an increased T and B lymphocyte population when compared with their young counterparts. This finding correlated with increased serum immunoglobulin level, known to be important in recruiting B lymphocyte in aged mice. SP reduced these excessive increased T and B lymphocyte and immunoglobulin level. Thus, the aim of the present study was to examine the effect of SP with respect to B cell dysfunction in aged mice, which leads to failure of Ig responses.

## Materials and Methods

### Preparation of SP Form *Bombyx mori*

SP, derived from the cocoons of *B. mori*, was obtained from Worldway Co., Ltd (Sejong, Korea). SP powder was prepared as follows: silkworm cocoons were acid-hydrolyzed for 24 h at 115–120°C and the solution was filtered. The filtrate was neutralized to pH 5.5–6.5 by addition of 33% NaOH and then re-filtered. After removing the salt, the solution was concentrated under vacuum (no <650 mmHg) at a temperature of 0 to 45°C. Finally, SP powder was obtained by spray drying the solution at 180 ± 10°C. The nutrient composition of the SP (100 g) was analyzed and the results are presented in Supplementary Data 1 in Supplementary Material.

The mean molecular weight of SP was measured using a MicroQ-time-of-flight (TOF) III mass spectrometer (Bruker Daltonics, Hamburg, Germany). Briefly, the sample was injected into an UltiMate 3000 high-performance liquid chromatography (HPLC) system (Dionex, CA, USA) fitted with a Poroshell 120 EC-C18 column (2.1 mm × 100 mm; 2.7 μm). The mobile phase comprised a mixture of acetonitrile and water (95:5 v/v). The flow rate was 200 μl/min. The results are shown in Supplementary Data 2 in Supplementary Material.

The free amino acid composition of SP was analyzed using an HPLC system comprising a pump (Waters 2695, Waters, Milford, MA, USA), an AccQ-Tag amino acid analysis column (Silica C18, 3.9 mm × 150 mm), and a Waters 2475 Multiλ Fluorescence detector (24, 25). The HPLC chromatogram is shown in Supplementary Data 3 in Supplementary Material. The crude biochemical composition of SP was determined according to the AOAC method, while pigments were analyzed using general methodologies (26).

### Animals and Treatments

Female C57BL/6 mice (aged 6 weeks or 12 months) were purchased from Joongah Bio (Seongnam, Korea). Mice were housed at 20 ± 3°C in a room maintained under a 12-h light–dark cycle. SP was dissolved in distilled water for oral administration. After 1 week of adaptation, aged (12 months) and young (6 weeks) mice received a daily oral dose (0, 100, or 750 mg/kg/day) of SP for 5 weeks. Body weight was measured once a week.

### Ethics Statement

All animals were treated humanely in accordance with the criteria outlined in the “Guide for the Care and Use of Laboratory Animals,” prepared by the National Academy of Science and published by the National Institutes of Health. All experiments were approved by the Institutional Animal Care and Use Committee (IACUC 180129) of CHA University (Seongnam, Kyunggi, Korea).

### Reagents

The following Abs and reagents were used in this study: FITC anti-mouse CD3ε (#100306), FITC anti-mouse CD19 (#152404), APC anti-mouse CD21/CD35 (#123412), PerCP/Cyanine5.5 anti-mouse CD23 (#101618), and Brilliant Violet 421 anti-mouse IgD (#405725) (all from BioLegend, CA, USA); PE anti-mouse CD45 (#12-0451-82) (Invitrogen, CA, USA); anti-TCR α/β (#sc-19600), anti-CD4 (#sc-19641), and anti-GAPDH (#sc-365062) (Santa Cruz Biotechnology, TX, USA); anti-MHC Class II (#ab180779), anti-ICOS (#ab175401), anti-ICOSL (#ab138354), anti-CD40 (#ab188181), and anti-CD40L (#ab2391) (Abcam, Cambridge, UK); and FITC anti-mouse CD169 (MOMA-1, #MCA947F) (Bio-Rad, CA, USA).

### Flow Cytometry Analysis of Cell Populations and Cell Sorting

Spleens obtained from C57BL/6 mice were homogenized and single-cell suspensions were prepared by passing the tissue through 40-μm cell strainers (SPL Life Sciences, Pocheon, Korea). The cells were then centrifuged for 5 min at 1,000 rpm and the supernatant was removed. Red blood cells were lysed with ACK lysis buffer (Lonza, Basel, Switzerland) (27). The cells were then washed again with PBS, centrifuged, and suspended in 100 μl of PBS. Finally, cells were incubated with the following anti-murine antibodies: CD3ε-FITC, CD19-FITC, CD45R-PE, CD21-APC, and CD23-PerCP-cy5. The cells were then washed twice with 500 μl of PBS and analyzed immediately by flow cytometry (FACSCalibur; BD Biosciences, Franklin Lakes, NJ, USA). Compensation and data analysis were performed using FlowJo software (Ashland, OR, USA). B and T cells from the spleen of young and old mice were sorted through MoFlo-XDP High Speed cell sorter (Beckman Coulter, CA, USA).

### Western Blotting

Splenocytes were lysed on ice for 15 min with ice-cold RIPA buffer [50 mM Tris-HCl (pH 7.4), 150 mM NaCl, 1 mM EDTA, 1% Triton X-100, 1% sodium deoxycholate, and 0.1% SDS] supplemented with protease inhibitors (1 mM PMSF, 5 μg/ml aprotinin, and 5 μg/ml leupeptin) and phosphatase inhibitors (1 mM Na_3_VO_4_ and 1 mM NaF) and then centrifuged for 5 min at 12,000 rpm at 4°C. The protein concentrations were measured in a BCA protein assay (Pierce, Rockford, IL, USA) using BSA as a standard. Proteins (20 μg/lane) were separated by SDS-PAGE and transferred to Immun-Blot PVDF membranes (Bio-Rad, Hercules, CA, USA). The membranes were incubated with 5% skim milk for 1 h at room temperature and then washed with Tris-buffered saline (TBS) containing 0.05% Tween-20 (TBS-T). The membranes were incubated overnight at 4°C with specific primary antibodies. After washing with TBS-T, the membranes were incubated for 1 h at room temperature with 5% skim milk containing peroxidase-conjugated anti-rabbit, anti-mouse, or anti-goat IgG. Signals were visualized using EZ-Western Lumi Femto (DoGenBio, Seoul, Korea) and quantified on a LAS-4000 (GE Healthcare Life Sciences, Marlborough, MA, USA). The intensity of the protein bands on each blot was quantified by densitometric analysis using ImageJ software (Bethesda, MD, USA) (28, 29).

### Isolation of RNA From Lymph Nodes and Quantitative Real-Time PCR (qRT-PCR)

Lymph nodes obtained from young and old C57BL/6 mice were homogenized and total RNA was extracted using Trizol reagent (Invitrogen, Carlsbad, CA, USA). Extracted RNA was reverse transcribed to cDNA using the Maxime RT PreMix kit (Intron, Seongnam, Korea). The sequences of the oligonucleotide primers were as follows: CD4, 5′-GAGAGTCAGCGGAGTTCTC-3′ (forward) and 5′-CTCACAGGTCAAAGTATTGTTG-3′ (reverse); IL-4, 5′-ACAGGAGAAGGGACGCCAT-3′ (forward) and 5′-GAAGCCGTACAGACGAGCTCA-3′ (reverse); ICOS, 5′-TCTGCCGTGTCTTTGTCTTCT-3′ (forward) and 5′-GAGCATTGGATTCTTGATGGA-3′ (reverse); ICOSL, 5′-ATCTCGTGGGGATGTTCTGT-3′ (forward) and 5′-GGTTTCCTGTGGGTTCTTTGT-3′ (reverse); OX40, 5′-GTAGACCAGGCACCCAACC-3′ (forward) and 5′-GGCCAGACTGTGGTGGATTGG-3′ (reverse); IL-21, 5′-GAAGATGGCAATGAAAGCCTGT-3′ (forward) and 5′-AGGATGTGGGAGAGGAGACTGA-3′ (reverse); and IL-21R, 5′-CCTTCTCAGGACGCTATGATATCTC-3′ (forward) and 5′-CTTGCCCCTCAGCACGTAGT-3′ (reverse). *Gapdh* was used as the control and qPCR was performed using Mx3005P qPCR System (Agilent Technologies, CA, USA).

### Measurement of Serum Cytokines and Immunoglobulins by ELISA

Blood was collected from the jugular vein of surviving mice at weeks 0, 2, 4, and 6 post-SP administration. Blood was centrifuged in a tabletop microcentrifuge for 10 min at 12,000 rpm. The supernatants were harvested, diluted 1:2, and the concentrations of IL-10, IL-13, and IL-6 in serum were analyzed using a LXSAMSM-06 kit (R&D Systems, MN, USA). To measure serum immunoglobulins, serum was collected from each mouse by cardiac puncture at the time of euthanasia. Sera were centrifuged in a tabletop microcentrifuge for 10 min at 12,000 rpm. The supernatants were harvested, diluted 1:25,000, and analyzed using a MGAMMAG-300K kit (Merck Millipore, MA, USA). All assays were performed according to the manufacturers' instructions. Cytokine and immunoglobulin levels in serum were measured using a Luminex 100 (Luminex, Austin, TX, USA).

### Immunohistochemistry (IHC)

Spleens were frozen in a mixture of dry ice–isopropanol and kept at −80°C until sectioning. Sections (10 μm) were cut on a cryostat, dried for 1 h at room temperature, and fixed for 10 min in acetone. The sections were then incubated with 0.1% BSA/PBS containing MOMA-1-FITC and anti-mouse IgD-Violet 421 mAbs prior to mounting in aqueous mounting solution and visualization under a Zeiss LSM880 confocal microscope (Zeiss, Oberkochen, Germany) fitted with a ×20 water immersion lens.

### MTT Assay

Splenocyte and purified B cells were seeded in 96-well plates at a density of 5 × 10^3^ per well and incubated overnight prior to treatment with SP (0, 50, 100, or 200 μg/ml) for an additional 24 h. MTT reagent was then added for 3 h, supernatant was removed, and 100 μl of DMSO was added to extract the intracellular formazan. Cell viability was measured at 570 nm in a PowerWaveHT ELISA reader (BioTek, Winooski, VT, USA).

### Statistical Analysis

All data are presented as the mean ± SEM. Statistical comparisons were made using Student's *t*-test or one-way analysis of variance (ANOVA) followed by Tukey's multiple range test. All statistical analyses were performed using SPSS (IBM, Armonk, NY, USA). A *p*-value < 0.05 was considered to indicate a statistically significant difference.

## Result

### SP Reduces the Increased T Cell Numbers in Old Mice

**Figure 1:**
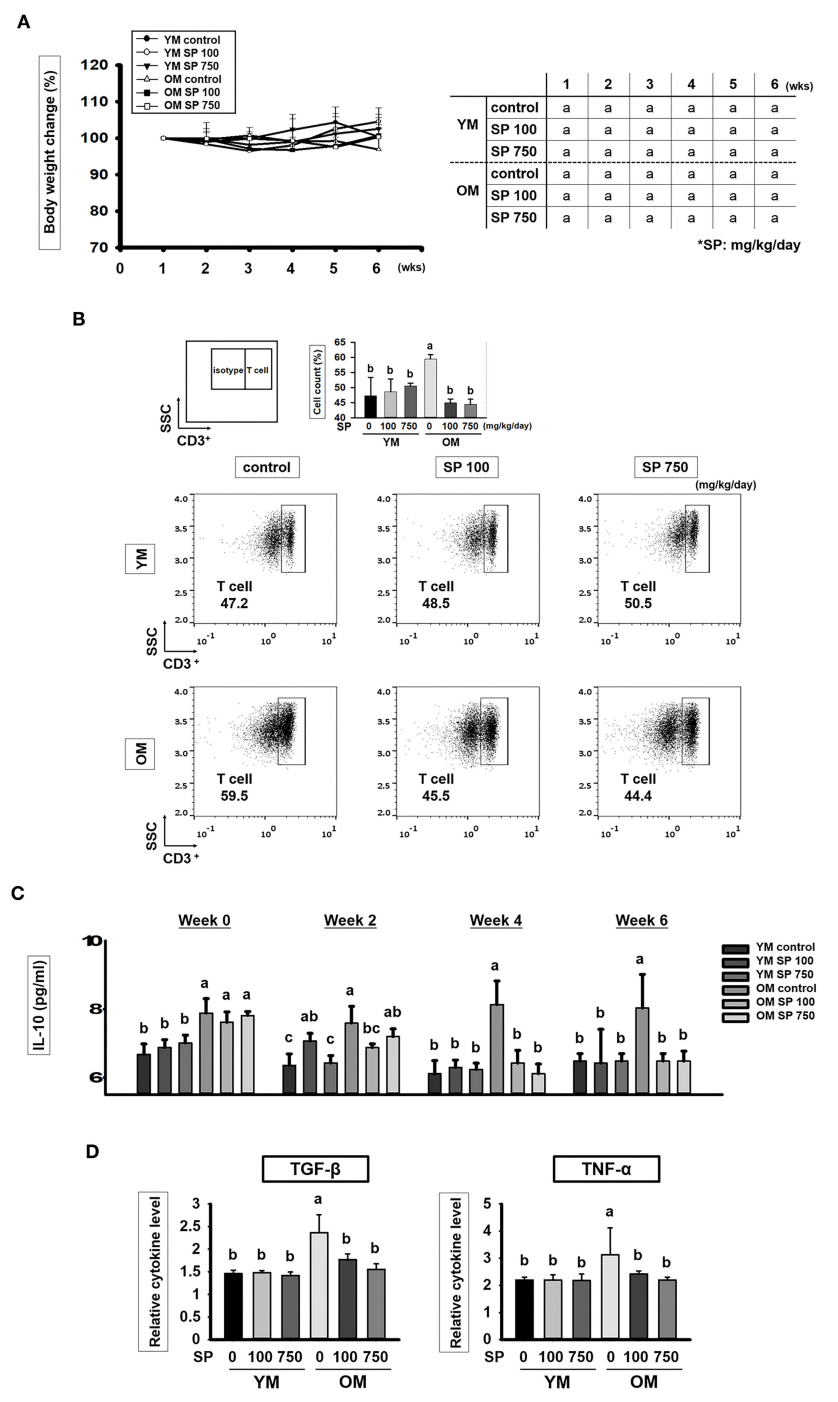
SP reduces the CD3+ T cell population and cytokines levels in old mice. **(A)** Percentage body weight change (relative to initial body weight at week 1) (mean ± SEM; *n* = 5). Mice (2 months old and 14 months old) were assigned to groups and orally administered SP (100 mg/kg/day or 750 mg/kg/day) for 5 weeks. **(B)** Splenic cells were stained using anti-CD3ε-FITC antibody. In the dot plots, CD3+ T cells are gated electronically. Representative results from young and old mice are shown. The numbers inside the plots represent the percentage of each cell population (mean ± SEM.; *n* = 3). The fluorescence scales are logarithmic. **(C)** Concentration of IL-10 in serum from experimental mice, as measured in an ELISA at weeks 0, 2, 4, and 6 (mean ± SEM; *n* = 4). **(D)** Concentration of TGF-β and TNF-α in mice serum at week 6 (mean ± SEM; *n* = 4). Statistical significance was determined using one-way ANOVA followed by Tukey's *post-hoc* test. Datasets denoted by different letters are significantly different (*p* < 0.05).

Young (6 weeks) and old (12 months) mice received SP (100 or 750 mg/kg/day) daily for 5 weeks. Body weight was measured once a week. As shown in Figure 1A, there was no statistically significant difference in body weight changes between the control groups and groups of old or young mice receiving SP. We also asked whether SP affects immune cell populations as mice age. First, we examined the total number of T cells (CD3^+^ cells) (Figure 1B). We found that the total number of CD3^+^ cells within the splenocyte population from old control mice was markedly higher than that from young control mice. However, the number of total T cells in the splenocyte population from old mice receiving SP (100 or 750 mg/kg/day) was as low as that in three young mice groups. Next, we measured the amount of interleukin (IL)-10, which is released by helper T (Th) cells, in mouse serum at weeks 0, 2, 4, and 6 (Figure 1C). From 3 weeks post-oral administration, IL-10 levels in old SP-treated mice fell to levels observed in three young mice groups. In addition, we measured the amount of TGF-β and TNF-α in mouse serum at week 6 (Figure 1D). Serum from old mice contained more TGF-β and TNF-α than serum form young mice, but the levels in old mice orally administered SP for 5 weeks fell significantly. These data indicate that the SP did not cause phenotypic changes in T cells; rather, after at least 4 weeks, it effectively reduced the number of CD3+ cells in the splenocyte population and reduced the concentration of IL-10, TGF-β, and TNF-α in serum, both of which increase with age.

### SP Ameliorates Excessive Immune Responses Through T and B Cell Interaction in Old Mice

**Figure 2:**
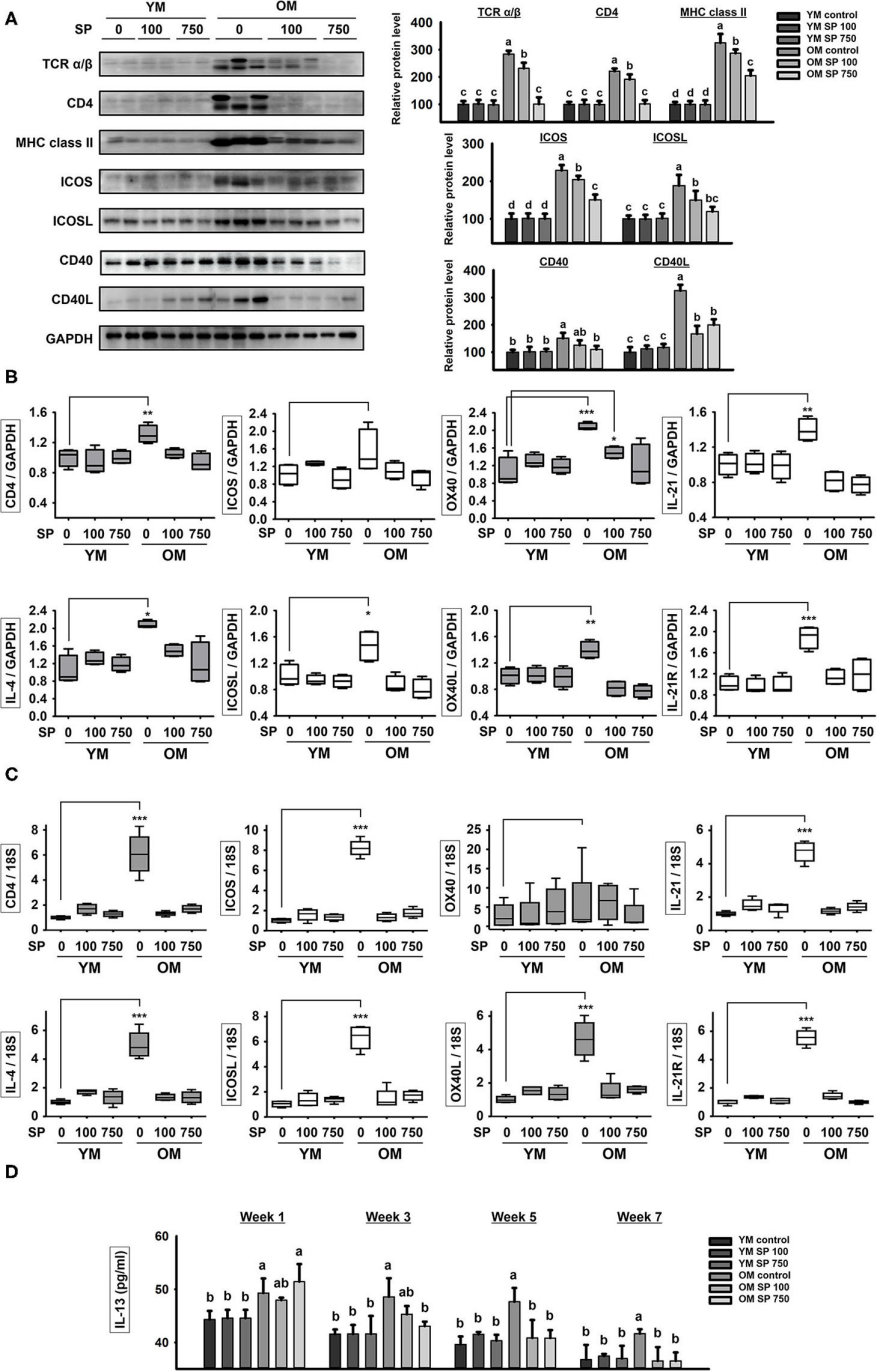
SP reduces expression of molecules associated with T cell/B cell interactions in old mice. **(A)** Western blot analysis of T/B cell interaction-associated molecules expressed by spleen cells obtained from experimental mice. GAPDH served as a control. **(B)** RNA was isolated from lymph nodes and quantitative RT-PCR analysis was performed to examine expression of mRNA encoding molecules associated with T/B cell interactions (mean ± SEM; *n* = 4). *p < 0.05, **p < 0.01, and ***p < 0.005, compared with the young mouse control group. **(C)** RNA was isolated from thymus and quantitative RT-PCR analysis was performed to examine expression of mRNA encoding molecules associated with T/B cell interactions (mean ± SEM; *n* = 4). *p < 0.05, **p < 0.01, and ***p < 0.005, compared with the young mouse control group. **(D)** IL-13 concentration in serum from experimental mice, as determined by ELISA at weeks 0, 2, 4, and 6 (mean ± SEM; *n* = 4). Statistical significance was determined using one-way ANOVA followed by Tukey's *post-hoc* test. Datasets denoted by different letters are significantly different (*p* < 0.05).

Next, we examined expression of costimulatory signaling markers expressed by T and B cells in the spleen (Figure 2A). TCR α/β, CD4, MHC class II, ICOS/ICOSL, CD40/CD40L, OX40/OX40L, and IL-21/IL-21R are surface markers that facilitate interaction between T and B cells. In the three groups of young mice, expression of all of these markers remained constant, regardless of SP treatment. However, expression of these markers was considerably higher in old control mice. After 5 weeks of oral SP, expression of all of these markers fell in a concentration-dependent manner. For genetic analysis, RNA was extracted from the lymph node and thymus of experimental mice and levels of mRNA encoding B cell and T cell interaction-associated markers (CD4, IL-4, ICOS, ICOSL, OX40, OX40L, IL-21, and IL-21R) were measured by quantitative RT-PCR (Figures 2B,C). Expression of mRNA for all of these genes was much higher in old control mice than in young control mice. Oral administration of SP for 5 weeks resulted in no differences in expression between young control mice and SP-treated old mice. Next, we measured the concentration of IL-13, which is released by T cells upon stimulation by B cells, in mouse serum at weeks 0, 2, 4, and 6 (Figure 2D). After 2 weeks of oral administration, SP reduced the amount of IL-13 in the serum of old mice to levels observed in three young mice groups. These results suggest that immune responses in young mice are unaffected by SP, whereas excessive age-related responses in old mice are ameliorated.

### SP Decreases the Total B Cell Population and Controls B Cell Subset Population in Old Mice

**Figure 3:**
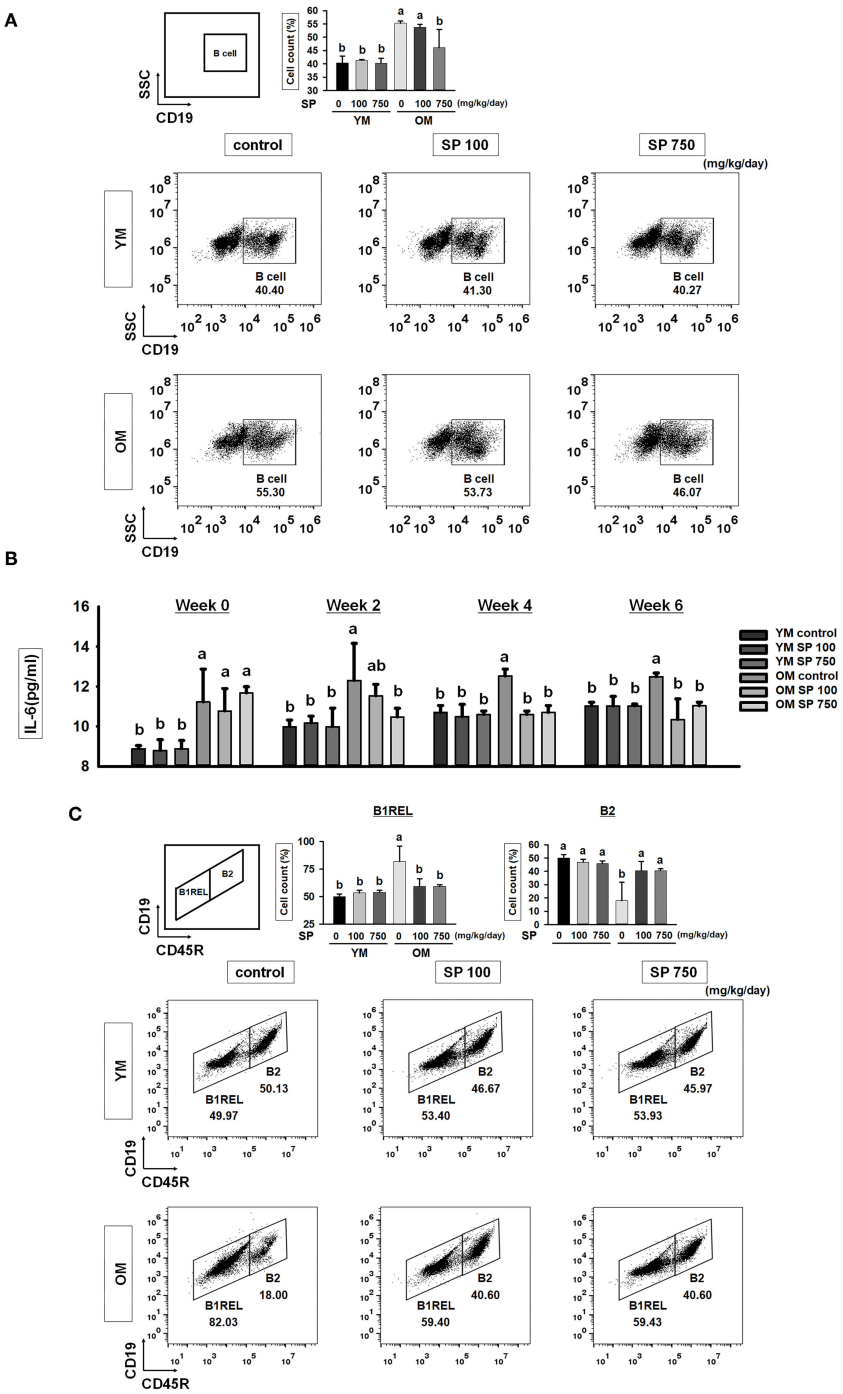
SP reduces the total B cell population and controls B cell subset populations in old mice. **(A)** Splenic cells were stained with anti-CD19-FITC. In the right-hand dot plots, CD3+ T cells were gated electronically. Representative data from young and old mice are shown. The numbers inside the plots represent the percentage of each cell population (mean ± SEM; *n* = 3). Fluorescence scales are logarithmic. **(B)** The concentration of IL-6 in serum from experimental mice, as determined by ELISA at weeks 0, 2, 4, and 6 (mean ± SEM; *n* = 4). **(C)** Cells were stained with anti-CD19-FITC and anti-CD45R-PE. The B1REL and B2 cell compartments were identified as CD19+ CD45Rlow (B1REL, left gate) and CD19+ CD45R+ (B2, right gate), respectively. Representative data from young and old mice are shown. The numbers inside the plots represent the percentage of each cell population (mean ± SEM; *n* = 3). The fluorescence scales are logarithmic. Statistical significance was determined using one-way ANOVA followed by Tukey's *post-hoc* test. Datasets denoted by different letters are significantly different (*p* < 0.05).

To investigate whether SP affects the total number of B cells in the splenocyte population in experimental mice orally administrated SP for 5 weeks, we examined expression of CD19 (a pan B cell surface marker). Flow cytometry analysis revealed a significant increase in the number of B cells within the splenocyte population from old mice compared with that in young mice (Figure 3A). However, in the SP 750 group of old mice, the number of B cells in the splenocyte population was reduced to the level observed in young mice. Next, we measured the amount of IL-6, which supports growth of B cells, in serum from experimental mice at weeks 0, 2, 4, and 6 (Figure 3B). Elevated levels of IL-6 were detected in the serum of old control mice. However, the high levels were reduced by SP. Next, we examined B cell subsets in the spleen. We measured the numbers of innate-like B1REL (CD19^+^ CD45R^low^) cells and B2 (CD19^+^ CD45R^+^) cells (Figure 3C). Compared with young control mice, old control mice had more B1REL cells. By contrast, the B2 cell population was considerably smaller. The size of these populations in the SP 100 and SP 750 groups of old mice was similar to that in young mice. Overall, these data show that SP regulates the number of B cells residing in the spleen, as well as some B cell subset populations, in old mice.

### SP Regulates the B2 Subset and Reduces Immunoglobulin Production in Old Mice

**Figure 4:**
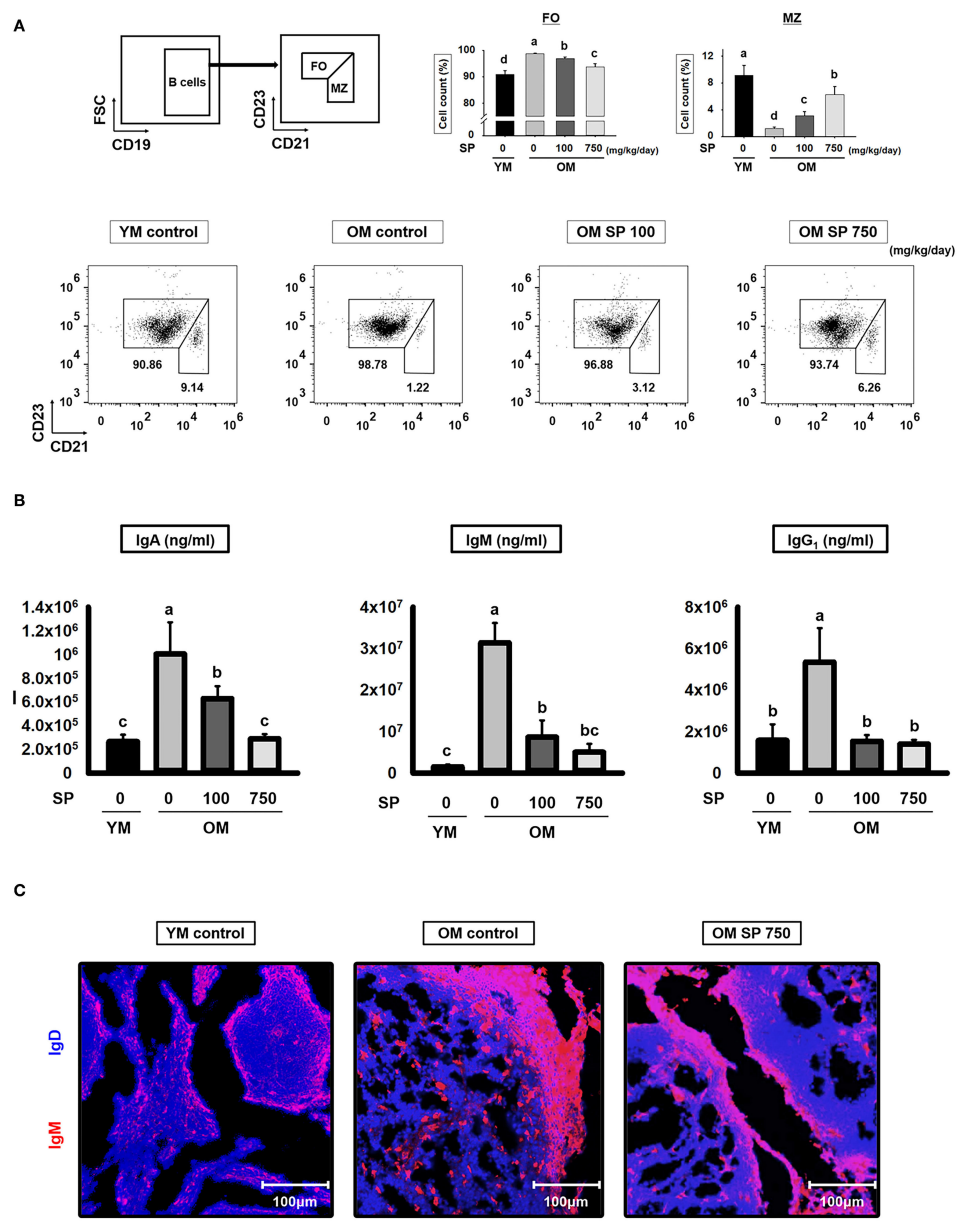
SP regulates FO and MZ B cells and reduces immunoglobulin production in old mice. **(A)** Splenic cells were stained with anti-CD21-APC and CD23-PerCP-cy5. The FO and MZ B cell compartments were identified as CD21int CD23high (FO B cell, left gate) and CD21high CD23low (MZ B cell, right gate), respectively. Representative data from young and old mice are shown. The numbers inside the plots represent the percentage of each cell population (mean ± SEM; *n* = 3). The fluorescence scales are logarithmic. **(B)** Serum was collected from experimental mice at the time of euthanasia and levels of IgA, IgM, and IgG1 were measured by ELISA (mean ± SEM; *n* = 5). **(C)** Immunofluorescence analysis of spleen sections from the indicated samples. Two-color staining was performed using IgM-Alexa647 (red) and anti-mouse IgD-Violet 421 (blue) mAbs. Representative photos from each group are shown. Statistical significance was determined using one-way ANOVA followed by Tukey's *post-hoc* test. Datasets denoted by different letters are significantly different (*p* < 0.05).

B cell development in the spleen results in two mature subsets: FO and MZ B cells. We performed flow cytometry analysis to investigate the effects of SP on FO and MZ B cells. Splenic B cells from experimental mice were separated into FO and MZ B cell populations according to surface expression of CD21 and CD23 (Figure 4A). FO and MZ B cells exhibit characteristic surface expression patterns: CD21^intermediate(int)^ CD23^high^ and CD21^high^ CD23^low^, respectively. Interestingly, the percentage of CD21^int^ CD23^high^ FO B cells in spleens from old control mice was significantly higher than that in spleens from young control mice. Reciprocally, the proportion of CD21^high^ CD23^low^ MZ B cells was lower in old control mice. After SP treatment for 5 weeks, the number of FO and MZ B cells in old mice fell in a dose-dependent manner. Since the main function of FO and MZ B cells is to produce immunoglobulins, we next examined the effect of SP on immunoglobulin levels in serum from experimental young and old mice (Figure 4B). Serum from old mice contained more immunoglobulin than serum from young mice; however, the levels in old mice orally administered SP fell markedly, ultimately reaching levels similar to those in young mice. To visualize the FO and MZ B cells in the spleen of experimental mice, we performed immunofluorescence analysis of tissue sections using IgD and IgM, as a marker of FO and MZ B cells, respectively (Figure 4C). The expression of IgM in control old mice was higher than that in young mice; however, the expression of IgM in SP-treated mice decreased.

### SP Reduces the Thickness of the MOMA-1 Layer and Expression of Apoptosis-Related Genes

**Figure 5:**
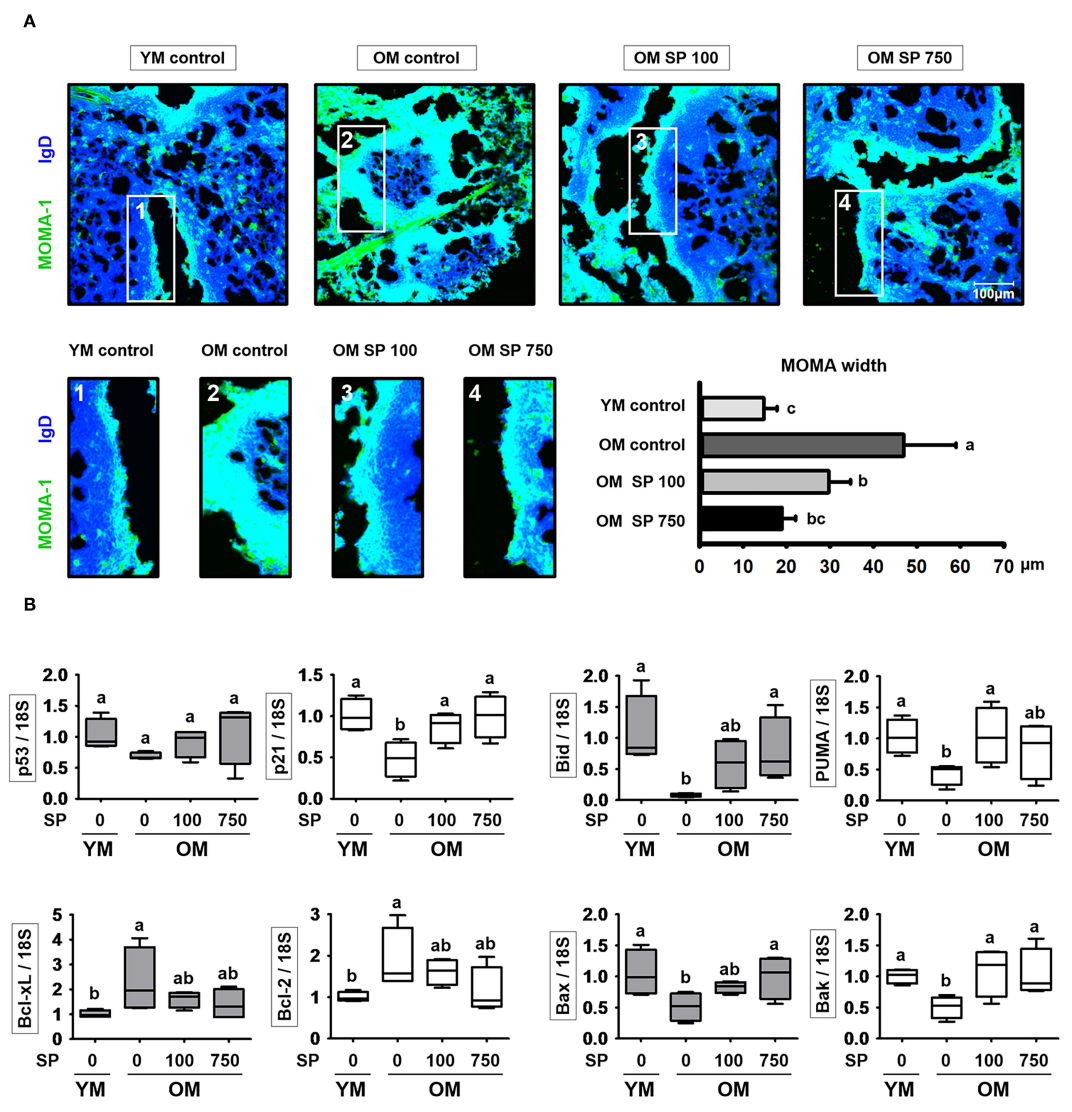
SP regulates phenotypic changes in the MOMA-1 layer and affects expression of apoptosis-related genes in the lymph nodes. **(A)** Immunofluorescence analysis of spleen sections from the indicated samples. Two-color staining was performed using MOMA-1-FITC (green) and anti-mouse IgD-Violet 421 (blue) mAbs. Representative photos from each group are shown. The boxes drawn in white indicate the regions magnified in the boxes below, i.e., the MOMA band around the follicles. Scale bars = 100 μm. The graph depicts the thickness of the MOMA band (μm). Band width was calculated from four different measurements per band. **(B)** RNA was isolated from lymph nodes and quantitative RT-PCR analysis performed to assess expression of cell death-related genes (mean ± SEM; *n* = 4). Statistical significance was determined using one-way ANOVA followed by Tukey's *post-hoc* test. Datasets denoted by different letters are significantly different (*p* < 0.05).

Finally, we asked whether the observed changes in splenic B cell numbers is indicative of changes in follicle organization. To visualize the MZ in the spleen of experimental mice, we performed immunofluorescence analysis of tissue sections using a MOMA-1 mAb as a marker of metallophilic macrophages (Figure 5A). The MOMA-1 band in control old mice was thicker than that in young mice; however, the depth of the MOMA-1 band in SP-treated mice decreased in a concentration-dependent manner. Previous studies show that an unorganized and thickened MOMA band affects B cell viability due to dysregulated apoptosis (30). Therefore, we collected RNA from the lymph node of experimental mice and examined the level of transcripts related to cell death using quantitative RT-PCR (Figure 5B). Expression of p21 and p53, both of which are associated with cell cycle arrest, was lower in old mice than in young mice; however, we observed a dose-dependent increase in expression in old mice orally administrated SP. Indeed, expression approached that in young mice. In addition, we measured expression of Bcl-2 family genes (including the anti-apoptotic members Bcl-2 and Bcl-xL), pro-apoptotic effector genes (Bax and Bak), and pro-apoptotic BH3-only genes (Bid and PUMA) (Figure 5B). Expression of pro-apoptotic genes, which was lower in old control mice than in young control mice, increased in old mice orally administered SP. By contrast, expression of anti-apoptotic genes, which was higher in old control mice than in young control mouse, diminished in SP-treated old mice. These results indicate that aging disrupts the organization of the MOMA band in the spleen, which may affect cell death by preventing normal apoptosis; however, this is mitigated by SP.

### SP Suppresses Excessive Expression of Surface Markers in T and B Cell of Old Mice *in vitro*

**Figure 6:**
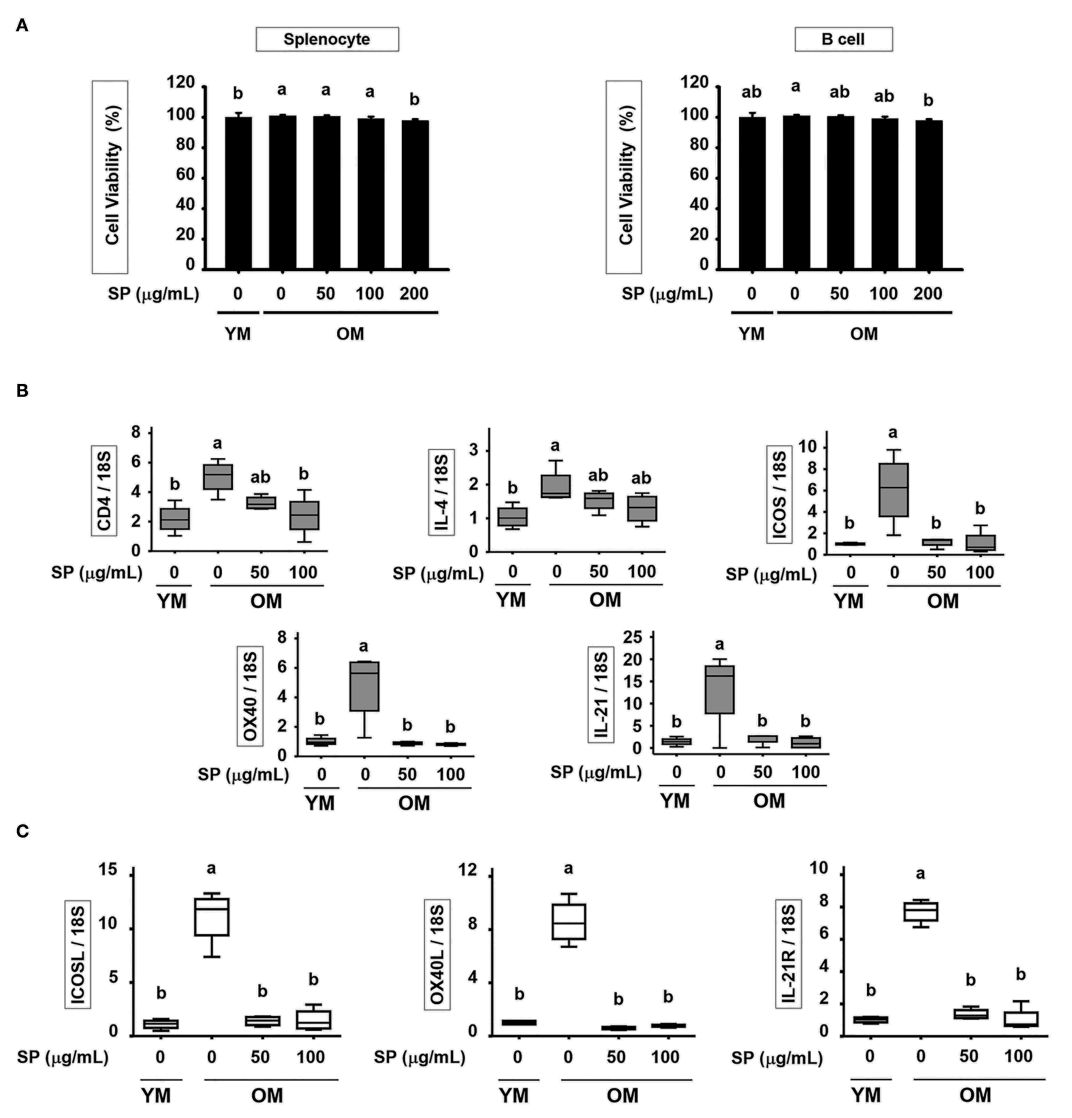
SP suppresses excessive expression of surface markers in T and B cell of old mice *in vitro*. **(A)** Splenocyte and isolated B cell were treated for 24 h with 0, 50, 100, or 200 μM SP in medium containing 1% serum. Cell viability was measured in an MTT assay after 24 h (mean ± SEM; *n* = 5). RNA was isolated from **(B)** T cells and **(C)** B cells, and quantitative RT-PCR analysis performed (mean ± SEM; n = 4). Statistical significance was determined using one-way ANOVA followed by Tukey's *post-hoc* test. Datasets denoted by different letters are significantly different (*p* < 0.05).

To determine the cytotoxic effect of SP *in vitro*, we treated splenocyte and purified B cells with various concentrations of SP (0, 50, 100, and 200 μM) for 24 h. In both splenocyte and B cell, cell viability was not significantly affected by SP until 100 μM (Figure 6A). In addition, we examined the levels of mRNA encoding B and T cell surface markers in purified B and T cell from spleen of young and old mice. Purified B and T cells were cultured with or without SP (50 or 100 μg/ml) for 18 h. Total RNA was extracted from the B and T cells and reverse transcribed to cDNA. The gene expression levels of CD4, IL-4, ICOS, OX40, and IL-21 in T cell and ICOSL, OX40L, and IL-21R in B cell were examined. The expression of all of these markers was considerably higher in old control compared to young control. However, SP treatment reduced expression of mRNA encoding these genes to levels observed in the young control group (Figures 6B,C).

## Discussion

As people age, immune homeostasis plays a crucial role not only in the innate immune system but also in the adaptive immune system (31). Here, we used 6-week-old (young) and 12-month-old (aged) mice to examine age-related changes in immunity, which is referred from (32). Immune system aging is characterized by underreacting or overreacting to small stimulations such as self-antigen like cellular proteins, peptides, and enzyme complexes, causing the immune homeostasis to break. In this research, we studied whether SP administration maintained a balance of immune homeostasis in the spleen, thymus, and lymph node (which are the representative adaptive immune organs) of 14-month-old mice as much as in normal physiological conditions in young mice. We found that treatment with SP restored homeostasis to the aged immune system by regulating the numbers and activity of T and B cells.

Initially, we showed that the number of CD3^+^ T cells in the splenocyte population from young control (untreated) mice was much lower than that from old control mice (Figure 1B). However, the numbers in old mice fell markedly after treatment with SP. We then found that after 4 weeks of treatment, SP also reduced the amount of IL-10, which is released by helper T cells, in old mice. Indeed, after 4 weeks, levels were similar to those in young mice (Figure 1C). Thus, SP restores homeostasis in old mice. Taken together, these data suggest that SP effectively down-regulates the number of total T cells in the spleen and the number of helper T cells, both of which increase due to aging. The latter (antigen-specific helper T cells) are important because they interact with antigen-specific B cells (5).

Next, we examined expression of costimulatory markers associated with interactions between T and B cells in the spleen (Figure 2A). Follicular B helper T cells (T_FH_) are CD4^+^ T cells that assist FO B cells located in secondary lymphoid tissues such as the lymph nodes and spleen (33). T_FH_ cells show characteristically high expression of CXC-chemokine receptor 5 (CXCR5). CXCR5 expression stimulates T_FH_ cells to migrate to the germinal centers where they stabilize their phenotype by making contact with local B cells via TCRα/β/CD4/MHC class II binding, ICOS/ICOSL binding, and CD40/CD40L binding (34–37). These interactions regulate proliferation and maturation of B cells and induce T_FH_ cells to secrete IL-4 and IL-21 cytokines (38). We found that T_FH_ interaction markers such as TCRα/β, CD4, MHC class II, ICOS, ICOSL, CD40, and CD40L were expressed at higher levels by splenocytes from old control mice than by those from young control mice. After 5 weeks of SP treatment, expression of T_FH_ interaction markers in old mice fell in a concentration-dependent manner; ultimately, levels were not significantly different from those in young mice (Figure 2A). In addition, expression of mRNA encoding T_FH_-associated markers in the lymph node was measured by quantitative RT-PCR; mRNA expression mirrored that of the proteins (Figure 2B). T_FH_ cells secrete IL-13, which directs the class switching of B cells from IgG1 (39). After 2 weeks of SP treatment, old mice showed decreased levels of IL-13 in serum; indeed, these fell to levels similar to those observed in young mice. These results suggest that SP maintains immune homeostasis by ameliorating the age-associated increase in T_FH_ numbers.

We wondered whether SP affects the B cell populations within splenocytes from old and young mice; therefore, we analyzed these cell populations by flow cytometry. The number of total B cells (CD19^+^) increases with age (15). However, we found that the percentage in old mice receiving 750 mg/kg/day SP fell to levels observed in young mice. In addition, the levels of IL-6, which supports the growth of B cell and causes disease when excessively produced by B cells (40, 41), fell to levels observed in young mice (Figures 3A,B). Innate-like B1REL cells are related to B1 cells and their splenic progenitors (42). Therefore, we examined the numbers of innate-like B1REL (CD19^+^ CD45R^low^) cells and conventional B2 (CD19^+^CD45R^+^) cells (42, 43). Compared with young control mice, old control mice harbored greater numbers of B1REL cells; by contrast, the B2 cell population was considerably lower (Figure 3C). B2 cells comprise FO and MZ B cells, which are found mainly in the spleen. The number of MZ B cells falls significantly as mice age (44). In addition, the aged splenic environment reduces migration of immature and MZ B cells (45). Therefore, we performed flow cytometry analysis to examine the effect of SP on MZ B cells. After SP treatment for 5 weeks, the number of CD21^high^ CD23^low^ MZ B cells in old mice increase to levels observed in young mice. This effect was dependent on the dose of SP. However, the numbers in non-treated older mice were lower than those in young mice (Figure 4A). Innate-like B1REL cells, which reside in the BM and spleen, produce 80% of the natural IgA, IgM, and IgG antibodies in mice (18, 46). Natural IgA, IgM, and IgG are polyreactive, low-affinity immunoglobulins that play roles in infection, B cell homeostasis, and auto immunity (19, 46, 47). Therefore, we asked whether SP affects the levels of these immunoglobulins. We found that SP reduced the increased IgA, IgM, and IgG levels in old mice (Figure 4B). IgA and IgM are important for both clearance of pathogens and removal of apoptotic cells (19). IgM produced by B-1 cells is essential for elimination of excess autoantigens, the number of which increases with age (19). We asked whether SP affects apoptosis during aging by examining the MOMA band in the spleen, along with expression of genes associated with apoptosis in the lymph nodes. The MOMA band, which is a metallophilic macrophage marker, affects B cell viability and apoptosis (30). The MOMA band in old control mice was thicker than that in young control mice. SP reduced the thickness of the MOMA band in aged mice (Figure 5A). In addition, we found that SP regulated expression of genes associated with cell cycle arrest and apoptosis (both pro- and anti-apoptotic genes) in mouse lymph nodes (Figure 5B). These results indicated that SP affects MOMA band organization and apoptosis in aged mice.

In conclusion, SP restores age-related declines in immune homeostasis. Age-related increases in T cell numbers (and the cytokines secreted by these cells) were reduced by SP. In addition, SP regulated the interaction between T and B cells, and certain B cell subset populations, in aged mice. Indeed, immunoglobulin levels in old mice were reduced to levels observed in young mice. Finally, aging disrupts the organization of the MOMA band in the spleen, which may affect apoptosis of senescent cells, thereby preventing their removal. SP mitigated MOMA band to affect senescent cell apoptosis. Taken together, these data suggest that SP strengthens the immune system in aged mice.

## Data Availability Statement

The raw data supporting the conclusions of this article will be made available by the authors, without undue reservation.

## Ethics Statement

The animal study was reviewed and approved by the Institutional Animal Care and Use Committee (IACUC 180129) of CHA University (Seongnam, Kyunggi, Korea).

## Author Contributions

SC and B-YL: conceptualization. H-JO: methodology and formal analysis. SC: validation and writing—original draft preparation. J-YL: resources and funding acquisition. SC and H-JO: writing—review and editing. HJ and KL: visualization. B-YL: supervision and project administration. All authors have read and agreed to the published version of the manuscript.

## Conflict of Interest

J-YL was employed by the company Worldway Co., Ltd. The remaining authors declare that the research was conducted in the absence of any commercial or financial relationships that could be construed as a potential conflict of interest.
