# Supplementary material for: Dysfunction of B Cell Leading to Failure of Immunoglobulin Response Is Ameliorated by Dietary Silk Peptide in 14-Month-Old C57BL/6 Mice
Source: Front Nutr. 2020 Nov 19;7:583186. doi: 10.3389/fnut.2020.583186 (PMC7710868; doi:10.3389/fnut.2020.583186)
Supplement: Supplementary file 1 [file Table_1.DOCX]

Supplementary Material

# Supplementary Figures and Tables

## Supplementary Figures


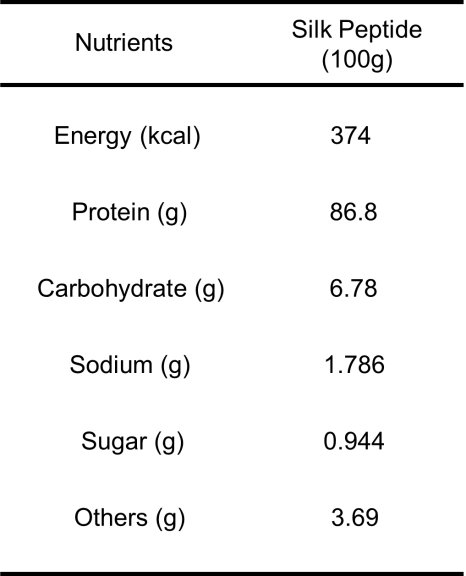


**Supplementary Figure 1.** The nutrient composition of the SP


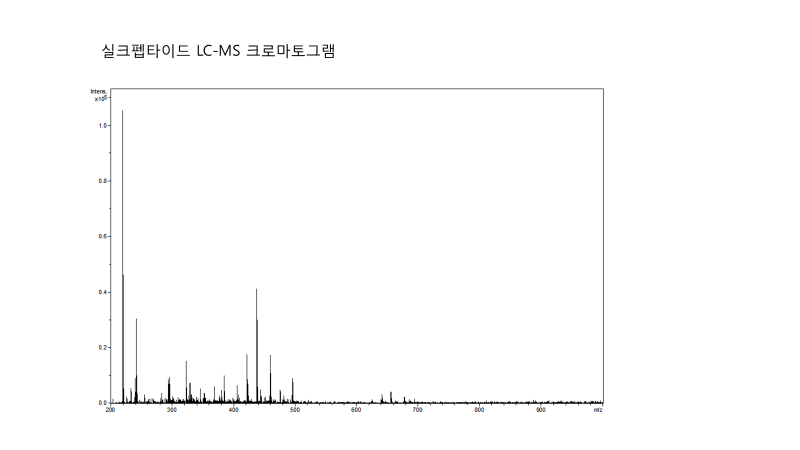


**Supplementary Figure 2.** Mass spectrometry analysis of Silk peptide. The figure shows that tandem mass spectrum generated by SP components.


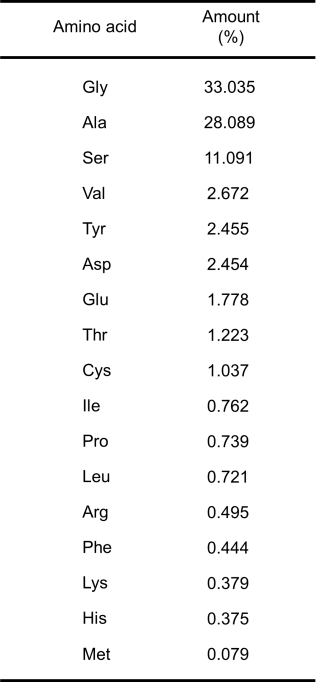


**Supplementary Figure 3.** Amino acid composition of the silk peptide, as assessed by high-performance liquid chromatography (HPLC). Abbreviations: Ala, Alanine; Cys, Cysteine; Asp, Aspartic acid; Glu, Glutamic acid; Phe, Phenylalanine; Gly, Glycine; His, Histidine; Ile, Isoleucine; Lys, Lysine; Leu, Leucine; Met, Methionine; Pro, Proline; Arg, Arginine; Ser, Serine; Thr, Threonine; Val, Valine; Tyr, Tyrosine; IS, Interpolation of standard (L-2-aminobutyric acid).
